# Application of Fe Based Composite Catalyst in Biomass Steam Gasification to Produce Hydrogen Rich Gas

**DOI:** 10.3389/fchem.2022.882787

**Published:** 2022-04-12

**Authors:** Liang Zhou, Zhiyong Yang, Deju Wei, Heng Zhang, Wei Lu

**Affiliations:** ^1^ School of Chemistry and Chemical Engineering, Guangxi University, Nanning, China; ^2^ School of Chemical Engineering, Guizhou Institute of Technology, Guiyang, China; ^3^ State Key Laboratory Breeding Base of Green Pesticide and Agricultural Bioengineering, Key Laboratory of Green Pesticide and Agricultural Bioengineering, State-Local Joint Laboratory for Comprehensive Utilization of Biomass, Ministry of Education, Center for Research and Development of Fine Chemicals, Guizhou University, Guiyang, China; ^4^ School of Mechanical Engineering, Guangxi University, Nanning, China

**Keywords:** biomass, gasification, Fe based catalyst, hydrogen rich gas, characterization

## Abstract

A series of composite catalysts with different Fe-based load amounts were prepared and applied to the experiment of biomass gasification assisted by steam. The structure of the catalyst was analyzed by XRD, SEM, TEM, N_2_ adsorption-desorption, and H_2_-TPR. The effect of the change of Fe load amounts on the catalytic activity was studied, and the optimal conditions of the gasification reaction were selected. The relationship between catalyst structure and catalytic capacity was clarified. The results showed that under the optimal reaction conditions, the catalyst showed better catalytic activity when Fe load amounts were 10%. The proportion of hydrogen in the gasification gas is as high as 42.2% and the hydrogen production is 27.65 g/kg. The tar content reaches the lowest value of 34.07g/Nm^3^.

## 1 Introduction

At present, global warming and energy supply security have become major strategic issues of common concern all over the world (United Nations, 2020). With the rapid and sustained growth of China’s economy, energy, resources, and environment have become serious constraints affecting future development. Vigorously developing renewable energy has important strategic significance (Wu et al., 2020; Huang et al., 2022). As a renewable energy, biomass is an important energy resource in China. It has played an important role in meeting energy demand, improving energy structure, reducing environmental pollution, and promoting economic and social development (Zhang et al., 2019a; Zhou et al., 2020). China is a largely agricultural country, which contains a lot of biomass energy, such as sawdust, straw, and firewood. Therefore, the development of biomass energy has broad prospects (Zhang et al., 2019b; Wu et al., 2021a; Choi et al., 2022).

Hydrogen energy has high combustion heat, no pollution, and wide sources, which are unmatched by traditional energy such as coal, oil, and natural gas (Ortiz and Gorri, 2021; Wu et al., 2021b). Gasification of biomass with the assistance of steam can produce hydrogen rich gas, which can be further processed and transformed to obtain chemical raw materials in short supply in China (Yang et al., 2020). In the gasification reaction, if there is a suitable gasification catalyst, it can not only improve the yield of hydrogen but also reduce the yield of tar produced by biomass gasification (Cortazar et al., 2021).

Common biomass gasification catalysts include Ni-based, alkali metal, mineral, and Fe-based catalysts (Demirba, 2002). Although the catalytic activity of Fe-based catalyst is not as good as that of Ni-based catalyst, its cracking ability of tar is comparable to that of calcined dolomite (Nordgreen et al., 2006). In addition, Fe-based catalysts have the advantages of low price, wide sources, and non-toxicity, which should be widely used. The oxidation states of Fe-based catalysts under different conditions are diverse, and Fe-based catalysts under different oxidation states have potential catalytic activity for biomass gasification.

Industrial wastes such as red mud, phosphogypsum, and coal gangue have almost no cost. Even after certain pretreatment, their price is much lower than that of traditional catalysts. In the field of biomass gasification, some scholars have studied red mud as a possible catalyst carrier (Wang et al., 2008; Li et al., 2021; Sun et al., 2021). Udomsirichakorn et al. (2013) applied quicklime (CaO) to steam gasification of pine sawdust in bubbling sludge bed and found that CaO has strong advantages in tar reforming and CO_2_ capture. Madhukar and maharishi (Mahishi and Goswami, 2007) studied that in a simple batch gasifier, CaO is used to increase H_2_ in the biomass gasification process and reduce CO_2_ content in the gas. In recent years, several studies have shown that when CaO is applied to biomass gasification experiments, it plays not only the role of carbon dioxide adsorbent but also the role of gasification catalyst (Tang et al., 2015; Zhou et al., 2019).

Therefore, in this study, the corresponding composites were prepared from industrial waste residue red mud, phosphogypsum, coal gangue, and CaO, and then the composites were modified with Fe salt to produce a series of catalysts with different Fe loading. The catalyst showed good catalytic activity in the process of biomass catalytic gasification to produce hydrogen rich gas. Combined with multiple detection methods, the effect of the change of Fe content in Fe-based composite catalyst on the catalyst activity was studied, and the optimal conditions of the gasification reaction were selected.

## 2 Experiment

### 2.1 Reagents and Instruments

The biomass raw material used in this experiment is pine sawdust, which comes from the Earth transportation mineral products processing plant in Lingshou County, China. Before the experiment, the biomass raw materials were dried at 105°C for 5 h. Before the gasification experiment, the biomass is mixed with the corresponding catalyst, a certain amount of sodium silicate solution is added, extruded, and granulated into particles <0.5, 0.5–1.0, and 1.0–1.5 mm. The industrial analysis and elemental analysis of pine sawdust are shown in Supplementary Table S1. Red mud comes from Guizhou Chinalco Group Co., Ltd., phosphogypsum from Guizhou kaiphosphorus Group Co., Ltd., and coal gangue from saping coal mine, Xiuwen County, Guizhou. Other reagents are analytical pure and purchased from Aladdin reagent company.

The instruments used in this experiment mainly include: collector constant temperature heating magnetic stirrer (DF-101s); Full automatic industrial analyzer (KDGF-8000A); Element analyzer (Elemental vario El/micro cube); X-ray fluorescence spectrometer (Bruker SRS3400); Gas chromatograph (Zhongke spectrum SP-7820); X-ray diffractometer (Rigaku Smart Lab); SEM (Tescan mira lms); TEM (FEI Tecnai G2 F20); Automatic specific surface and pore size distribution analyzer (Quantachrome Autosorb-iQ); Temperature programmed adsorption instrument (FINESORB 3010A).

### 2.2 Gasification Unit

**FIGURE 1:**
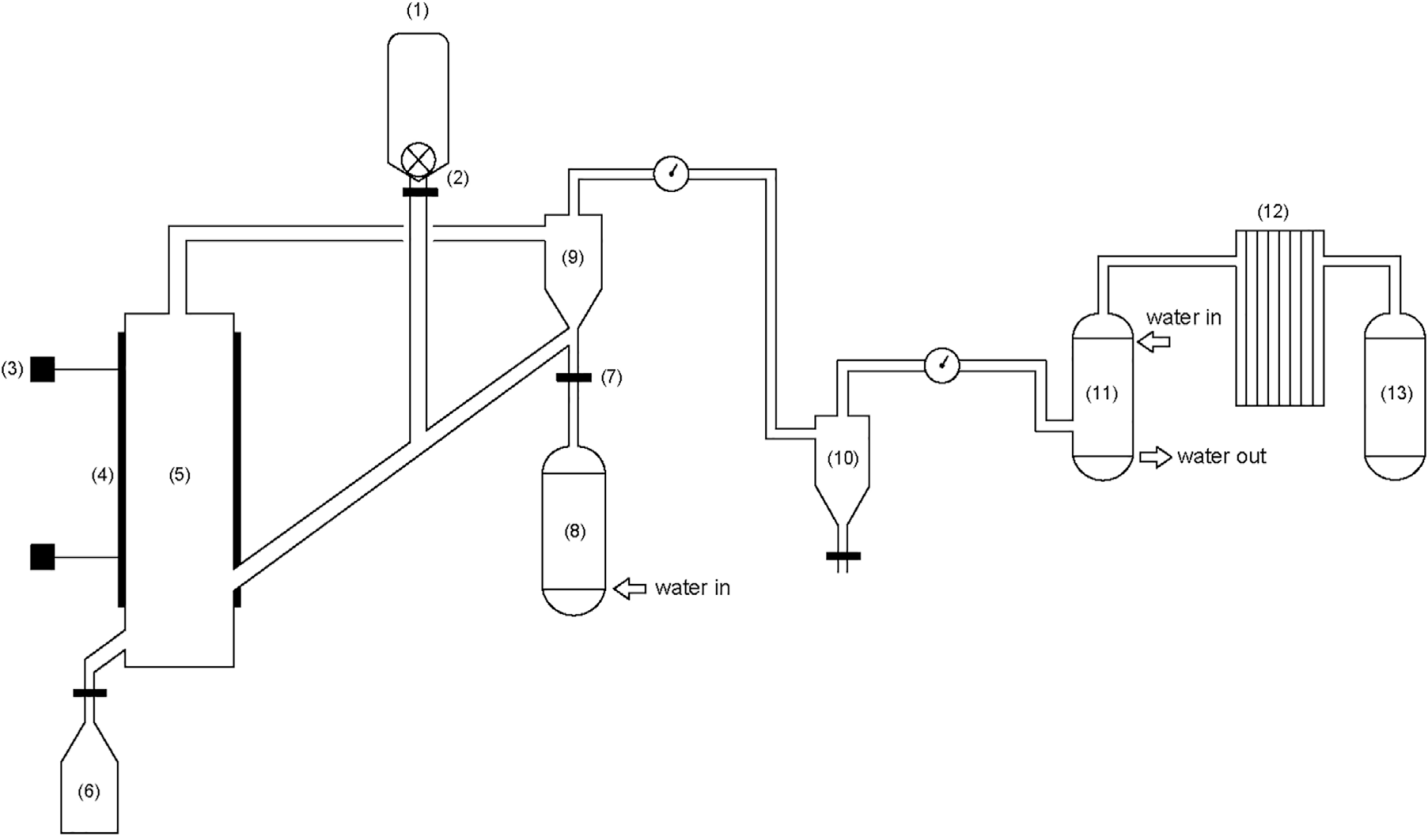
Fluidized bed biomass steam gasification system. 1) Biomass hopper; 2) Stepping feeder; 3) Thermocouple; 4) Heater; 5) Gasifier; 6) Ash hopper; 7) Mass flowmeter; 8) Boiler; 9) Primary cyclone separator; 10) Secondary cyclone separator; 11) Scrubber; 12) Filter tower; 13) Gas collection device.

The fluidized bed equipment (Figure 1) used in this gasification experiment is self-assembled and built. The experimental system is a circulating system (a large amount of inert gas is filled as circulating gas before work), which is mainly composed of three small systems: gasification system, purification system, and gas storage system from left to right.

### 2.3 Analysis of Products

#### 2.3.1 Qualitative and Quantitative of Gas Components

The volume of non-condensable gas is quantified by a wet flowmeter and then collected by a gas collection bag. The main components are H_2_, CO, CH_4_, CO_2_ which are analyzed by gas chromatography.

#### 2.3.2 Collect and Weigh the Tar and Char Produced in the Gasification

Use the reagent bottle containing acetone to recover the tar discharged from the lower part of the scrubber. After collection, mix excess anhydrous copper sulfate into the mixture and filter it to remove the water in the mixture. Pour the mixture into a 500 ml conical bottle and put it in a constant temperature water bath heater at 70°C until the acetone volatilizes completely and the residue is tar. The mass of tar is the mass of the heated conical bottle minus the mass of the empty bottle, which is calculated by the difference method.

Collect the solid produced in the experiment from the ash hopper and secondary cyclone separator. After deducting the amount of added catalyst, the remaining solid is weighed and marked as char.

### 2.4 Preparation of Fe Based Composite Catalyst

The chemical composition of red mud, phosphogypsum, and coal gangue is determined by the X-ray fluorescence spectrum. The chemical composition analysis is shown in Table 1.

**TABLE 1:** The chemical composition of industrial wastes (wt%).

Composition	SiO_2_	Al_2_O_3_	Fe_2_O_3_	CaO	MgO	K_2_O	Na_2_O	TiO_2_	SO_3_	P_2_O_5_
Red mud	18.35	24.27	15.12	16.48	1.98	1.81	4.55	2.12	0.34	0.19
Phosphogypsum	12.32	1.89	0.76	26.7	0.28	0.66	0.21	0.23	36.33	0.88
Gangue	55.3	26.4	7.58	1.3	1.25	0.81	1.60	2.44	0.68	0.13

#### 2.4.1 Preparation Method of Fe Based Catalyst

1) Preparation of red mud phosphogypsum slurry.

Dry, crush and grind the red mud and phosphogypsum respectively, screen and select 100 mesh raw materials. Weigh a certain amount of phosphogypsum and red mud respectively, and the mass ratio of the two is 3:7 for use. Phosphogypsum is mixed with water to form a suspension, and red mud is gradually added to make it a uniformly mixed slurry.

2) Preparation of composites.

Weigh according to the ratio of slurry (dry basis mass)/CaO mass ratio of 7/3. Weigh the crushed coal gangue (100 mesh), and its mass is 10% of the dry basis mass of the slurry. Weigh sodium carbonate, and the mass is 3% of the total mass of the first several compounds (dry basis). Mix the weighed slurry with crushed CaO and coal gangue evenly. Use a certain amount of sodium carbonate dissolved in water as the foaming agent, add it to the mixed raw materials, add water and stir evenly, put it into a 100°C oven for constant temperature drying for 24 h, remove the water, put it in a muffle furnace, calcine at 1,000°C for 5 h. And then it is broken and passed through a 100 mesh sieve to obtain particles for the next step.

3) Preparation of Fe based composite catalysts.

In order to make the loading amount (weight percentage) of Fe in the composite catalyst 5%, 10%, and 15% respectively, the amount of Fe (NO_3_)_3_·9H_2_O is calculated by using the mass of <0.5 mm composite particles prepared in the previous step. Weigh an appropriate amount of Fe (NO_3_)_3_·9H_2_O, prepare a solution with deionized water into a certain concentration, transfer the corresponding FeNO_3_ solution, soak the particles prepared in the previous step, stand for 24 h, and put it into a 100°C oven for constant temperature drying for 24 h to evaporate the water. Then put the dried mixture into the muffle furnace, calcine it at 1,000°C for 5 h. Let it cool naturally in the muffle furnace, take it out, break it and screen it into particles less than 100 mesh for use, named catalyst a, b and c respectively.

### 2.5 Catalyst Performance Evaluation

Gas yield, tar yield, char yield, tar content, gas composition H_2_, CO_2_, CO, CH_4,_ and H_2_ production are used as the criteria for evaluating the activity of the catalysts. The relevant calculation formula is as follows:

① Tar yield = tar mass/added absolute dry biomass raw material mass × 100%.
② Char yield = char quality/added absolute dry biomass raw material mass × 100%.
③ Gas yield = 100-Tar yield-Char yield.
④ Tar content (g/Nm^3^) = tar quality (g)/dry gas volume (Nm^3^).
⑤ H_2_ production (g/kg) = weight of H_2_ (g)/weight of biomass raw material (kg).

## 3 Results and Discussion

Biomass gasification process is complex. The reaction process is different with the different process flow, reaction conditions, gasification medium, raw material properties, and other conditions. However, the basic reactions of these processes include biomass gasification reaction, reduction reaction, tar conversion reaction, and so on.

Biomass→Tar + Char + Gas (H_2_、CO、CO_2_、CH_4_、C_n_H_m_)                      (R1)
Tar → CH_4_+H_2_O + C_m_H_n_ + H_2_             (R2)
CH_4_ + H_2_O → CO + 3H_2_              (R3)
C_n_H_m_ + nH_2_O → nCO+(n + m/2) H_2_         (R4)
C_n_H_m_ + nCO_2_ → 2nCO+(m/2) H_2_        (R5)
C + CO_2_ → 2CO                (R6)
C + H_2_O → CO + H_2_              (R7)
C+2H_2_O → CO_2_+2H_2_              (R8)
CO + H_2_O → CO_2_+H_2_                 (R9)
CaO + CO_2_ → CaCO_3_             (R10)

### 3.1 Effect of Catalysts With Different Fe Load Amounts on Gasification Reaction

**FIGURE 2:**
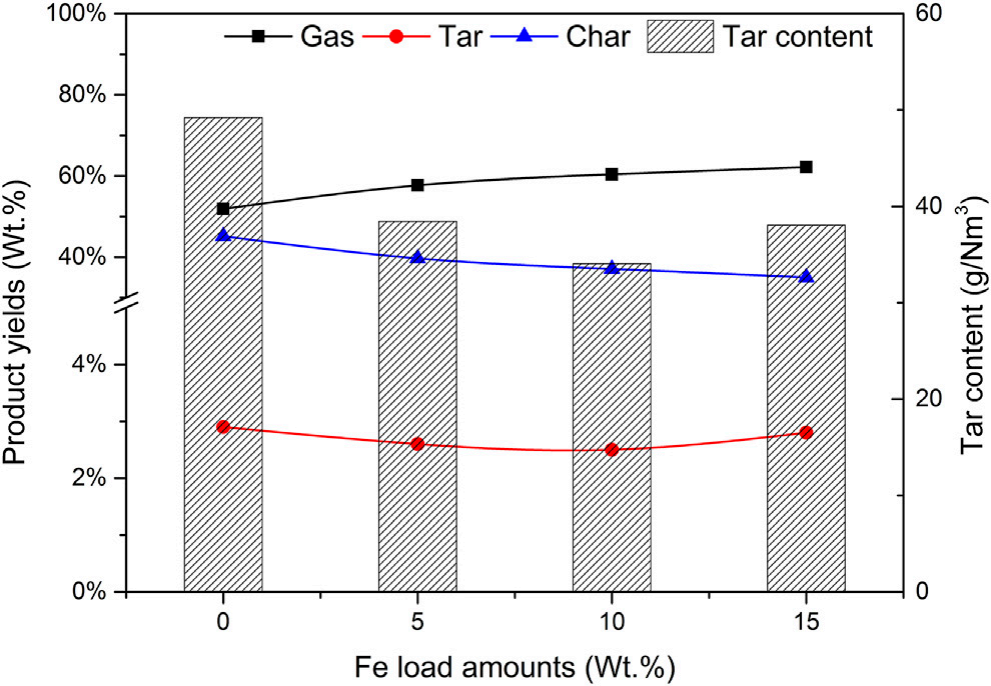
Effect of Fe load amounts on product yields and tar content.

The Fe-based composite catalyst can effectively catalyze the gasification reaction of pine sawdust. When the mass fraction of Fe load amounts are 0%, 5%, 10%, and 15% respectively, the activity evaluation results of the prepared Fe-based composite catalyst are shown in Figure 2. Other conditions of the reaction are as follows: the mass ratio of catalyst to biomass (CBR) is 1.2, the gasification reaction temperature is 650°C, the particle size of biomass is less than 0.5 mm, and the mass ratio of steam to biomass (SBR) is 1.0.

It can be seen from Figure 2 that with the increase of Fe load amounts, the gas yield increases gradually, and the yields of tar and char also change. When the mass fraction of Fe is 10%, the gas yield is reaching 60.4%, and the yields of tar and char are 2.5% and 37.1% respectively.

**FIGURE 3:**
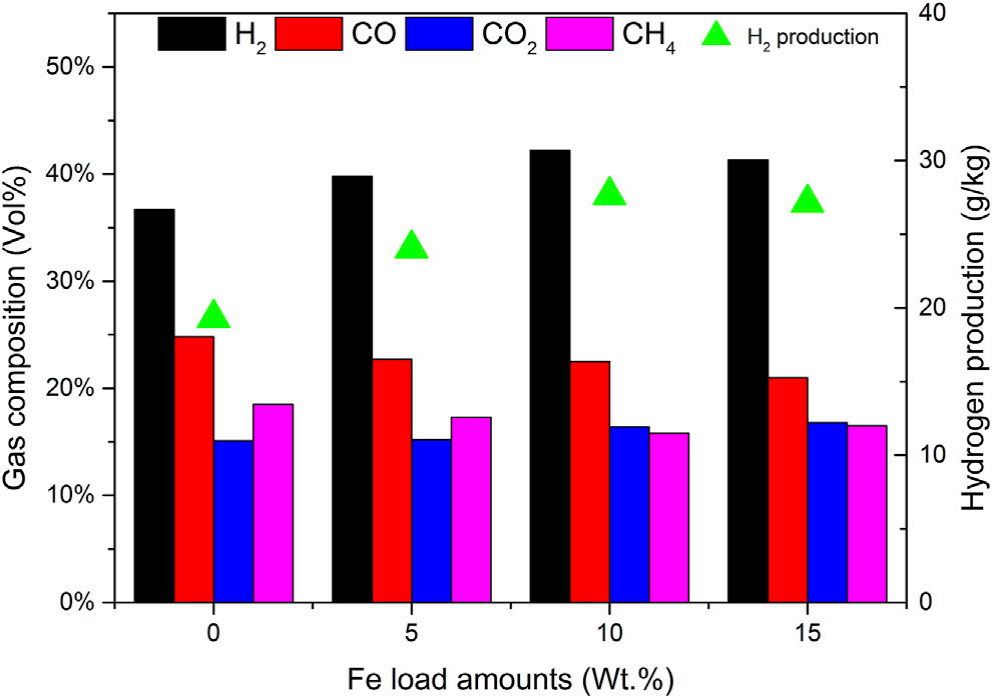
Effect of Fe load amounts on gas composition and hydrogen production.

Compared with composites with 0% Fe load and catalyst a, when the load amounts are low, the yields of tar and char are high. The reason is that the addition of the Fe base is conducive to tar reforming and char gasification. As the Fe load amounts increased to 15%, the percentage of H_2_ in Figure 3 did not increase, but decreased to 41.3%, so that the output of H_2_ also decreased. It does not seem that the higher the Fe load amounts, the better the catalytic efficiency. There is an optimal value in the experiment. The catalytic activities of catalysts a and c are significantly lower than those of catalyst b. Considering the catalytic efficiency and economic factors, the Fe-based loading of 10% is the most appropriate.

### 3.2 Characterization of Fe Based Composite Catalysts

#### 3.2.1 XRD Characterization

**FIGURE 4:**
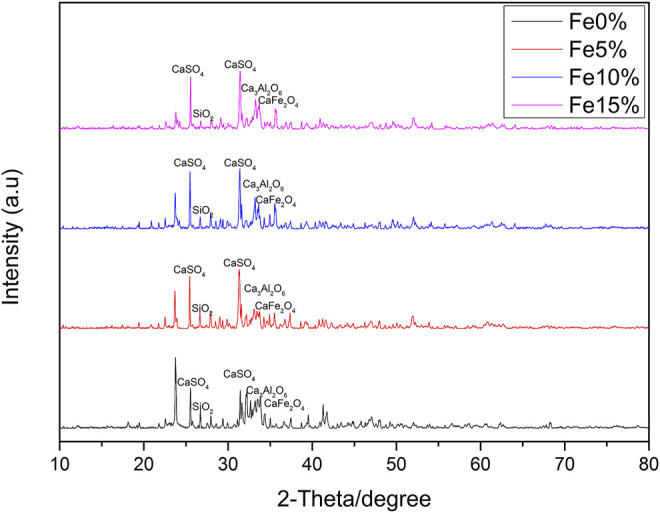
XRD spectra of Fe based composite catalysts with different Fe load amounts.

Figure 4 shows the wide-angle XRD spectrum of Fe-based composite catalysts a, b and c. There are many diffraction peaks in the figure, indicating that the crystal form in the sample is relatively complex, and its main chemical components are CaSO_4_, SiO_2_, Ca_3_Al_2_O_6_, CaFe_2_O_4,_ and so on. Among the catalysts prepared by the impregnation method, the diffraction peak intensity of CaFe_2_O_4_ particles in catalyst a is the lowest. When the load amounts of Fe element increases to 15%, the intensity of CaFe_2_O_4_ diffraction peak increases, and the peak width narrows, indicating that CaFe_2_O_4_ particles increase with the increase of load amounts, dispersion of particles decreases, aggregation occurs and the reaction activity becomes worse. The diffraction peak intensity of catalyst b is weaker than that of catalyst c, indicating that the supported Fe-based compounds are better dispersed on the composites. It can be seen that the catalytic activity of Fe-based composite catalyst is b > c > a, which is in a certain compliance relationship with the diffraction peak intensity of CaFe_2_O_4_.

#### 3.2.2 SEM Analysis

**FIGURE 5:**
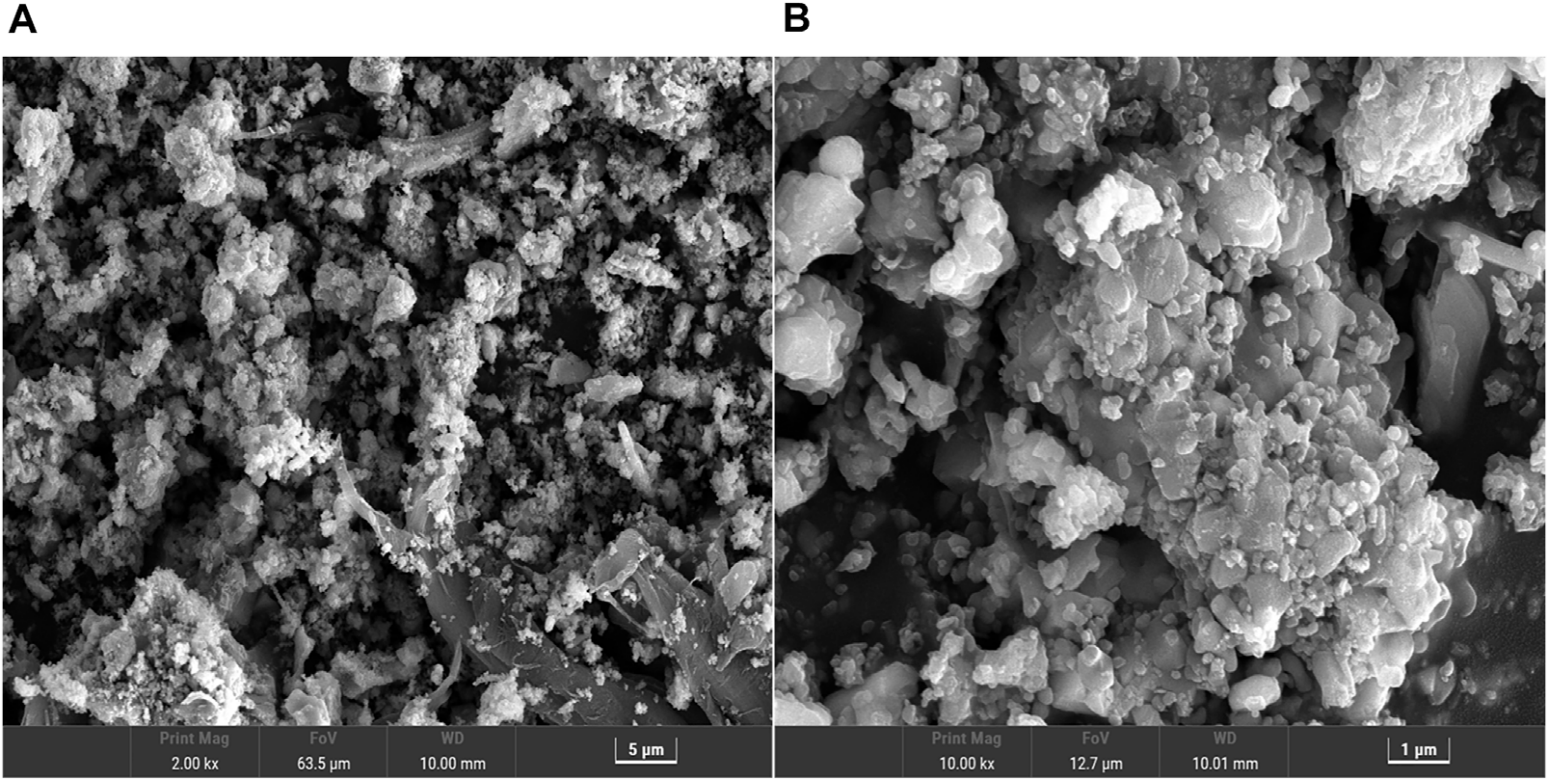
SEM of catalyst b: **(A)** magnified 2000 times **(B)** magnified 10,000 times.

The working principle of SEM is to use the electron beam generated by cold field emission to scan the material surface, detect the secondary electrons and backscattered electrons generated by the electron beam excited surface, and determine the micromorphology of the sample surface. For the complex and rough sample surface, a clear image can be obtained. The structure and morphology of the prepared 10% Fe-loaded catalyst b were analyzed by scanning electron microscope. Figure 5A is an enlarged picture of 2000 times. The figure shows that the catalyst is a nanomaterial with a diameter of about 200–500 nm, which is united and gathered by many irregular particles. This nanoscale structure makes the solid catalyst generally have a large specific surface area so that the active sites of the reaction can be fully exposed, which is conducive to improving the catalytic activity of the catalyst. Figure 5B shows the image with a higher magnification of 10,000 times. At the same time, it can be observed that there are many small sheets with irregular shapes in the sample, which are bonded layer by layer to form relatively dense particles.

#### 3.2.3 TEM Analysis

**FIGURE 6:**
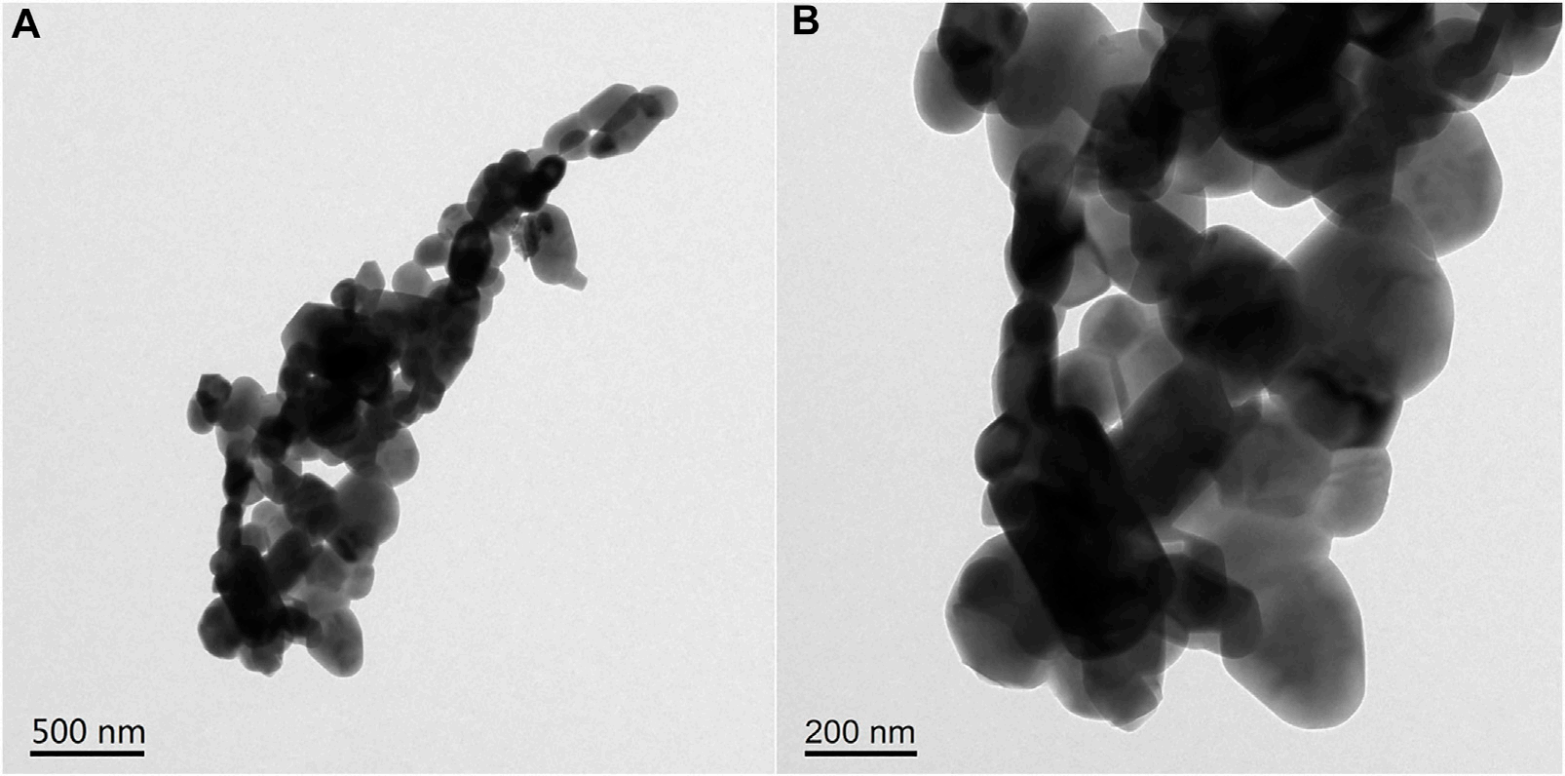
TEM of catalyst b: **(A)** 500 nm scale **(B)** 200 nm scale.

In order to further study the surface microstructure of catalyst b and verify the existence of its porous structure, catalyst b was characterized by TEM. The results are shown in Figure 6A (500 nm scale). It can be clearly observed that catalyst b is formed by stacking and agglomeration of crystal particles with different sizes and shapes. When further enlarged, as shown in Figure 6B (200 nm scale), it can be more clearly observed that the catalyst presents a vermicular disordered mesoporous structure. This porous structure may come from the ordered aggregation of materials. In addition, these connected holes are randomly connected, not orderly and regular linear shapes.

#### 3.2.4 BET Analysis

The surface characteristics of Fe-based composite catalysts a, b and c were evaluated by the N_2_ adsorption-desorption method. The results are listed in Table 2. It shows that the composite catalyst has a quite good specific surface area and pore structure. The high specific surface area and pore structure indicate that it may have good catalytic activity and the feasibility of being a high-efficiency catalyst. With the increase of Fe load amounts, the average pore size and pore volume of catalysts a, b and c have a downward trend. It is speculated that Fe-based species are mainly successfully attached to the pore surface of the composite, which is more conducive to full contact with reactants and has the potential characteristics of excellent catalysts. At the same time, it can be seen that the Fe load amounts increase, the pores become smaller, and the Fe-based species agglomerate. These phenomena show that too much load amounts are not conducive to the catalytic activity.

**TABLE 2:** Surface characteristics of Fe based composite catalyst.

Catalyst	Specific Surface area(m^2^/G)	Average Pore size(nm)	Pore volume(cm3/G)
a	229.5	537.2	0.40
b	225.3	519.6	0.35
c	220.1	484.5	0.33

#### 3.2.5 TPR Characterization

**FIGURE 7:**
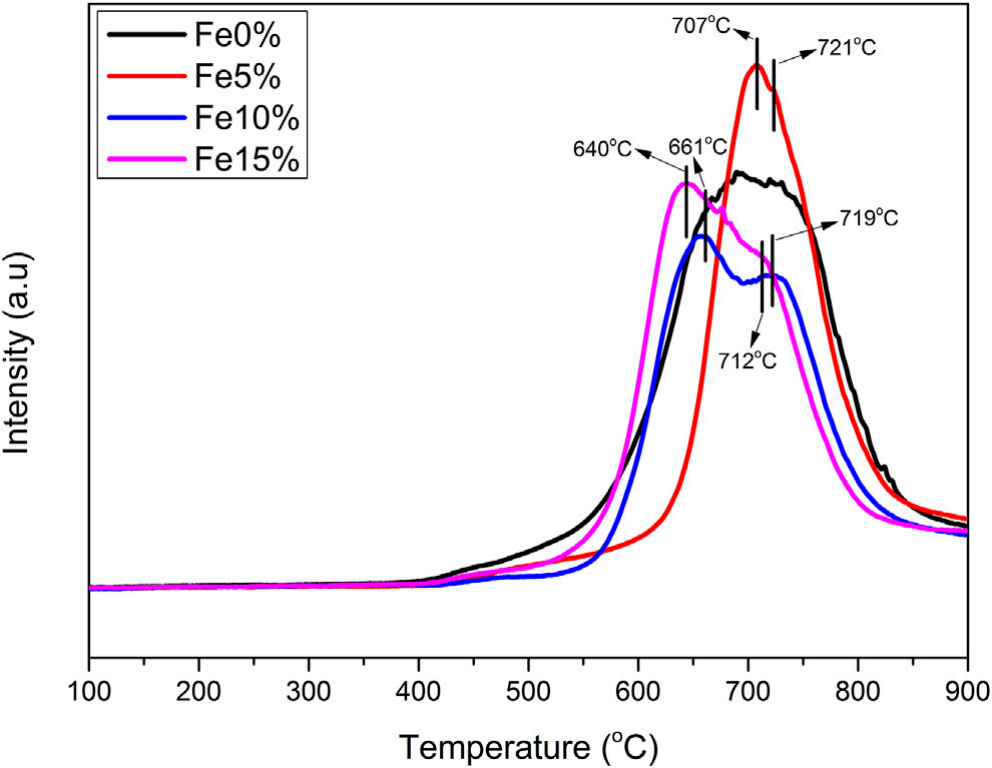
H_2_-TPR characterization.

Figure 7 shows the TPR curves of Fe-based composite catalysts a, b and c. The three composite catalysts have multiple H_2_ reduction peaks. The reduction peak between 640°C, 661°C, 707°C can be attributed to the reduction of CaFe_2_O_4_ → Fe_3_O_4_ in the sample. The high-temperature reduction peak is between 712°C, 719°C, 721°C, which can be attributed to the continuous reduction of Fe_3_O_4_ → FeO/Fe (Cabello et al., 2014a; Cabello et al., 2014b). By comparing the TPR curves of Fe based composite catalysts with different Fe load amounts, it can be seen that the high-temperature peak of the sample with large Fe loading moves to the low-temperature direction, which is due to the simple substance formed after the metal oxide in the catalyst is reduced, hydrogen is adsorbed on the surface, and the activated hydrogen species reach the surface of the composite catalyst through overflow, It promotes the reduction of various metal elements in the sample at a lower temperature.

### 3.3 Effect of Mass Ratio of Catalyst b to Biomass (CBR) on Gasification

The selection of CBR is very important to the gasification process. The gasification conditions were selected as follows: catalyst b, reaction temperature 650°C, biomass particle size <0.5 mm, and SBR = 1.0. The gasification effects of CBR 0.8, 1.0, 1.2, and 1.4 were investigated.

**FIGURE 8:**
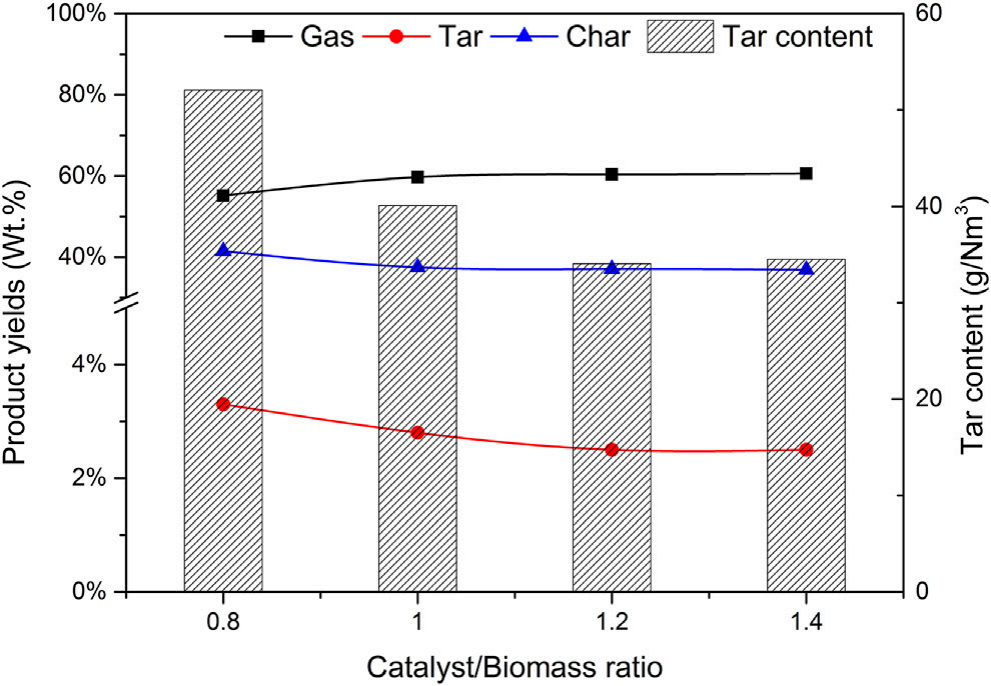
Effect of CBR on product yields and tar content.

Figure 8 shows the effect of CBR on each product component and tar content. During the increase of CBR from 0.8 to 1.4, the percentage of gas increases significantly, while tar and char are gradually decreasing. The higher the CBR value, the higher the gas yield. This phenomenon can be explained that the reaction R10 is an exothermic reaction. When CaO captures CO_2_, it will release heat, which may increase the temperature around biomass. This higher temperature is also conducive to the increase of tar cracking and char conversion. For the above reasons, when CBR = 1.2, the tar content reaches the lowest value of 34.07 g/Nm^3^.

**FIGURE 9:**
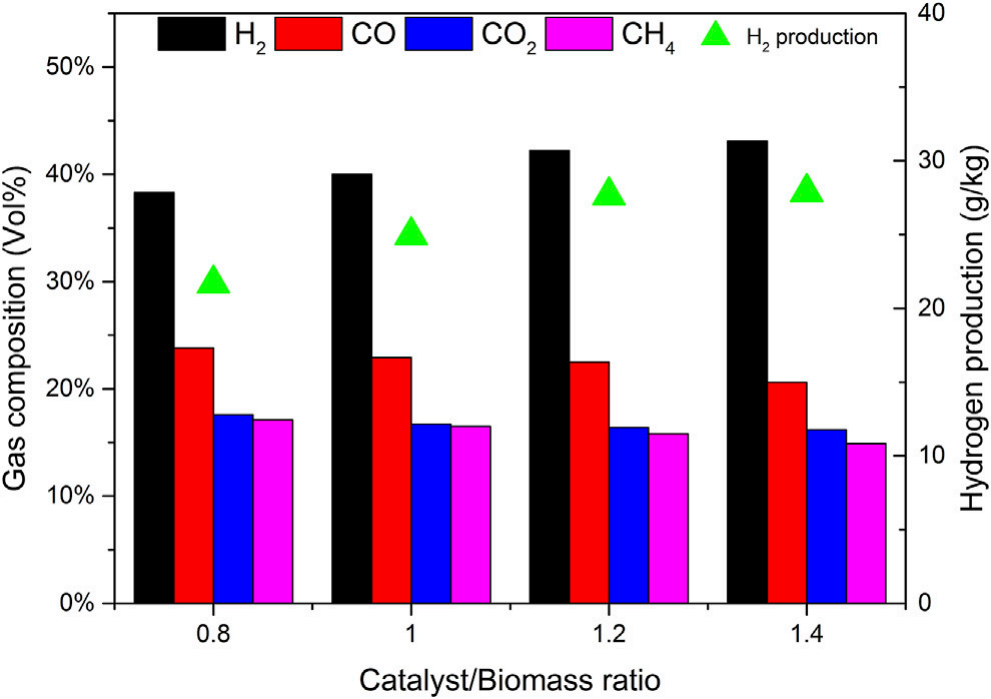
Effect of CBR on gas composition and hydrogen production.

Figure 9 shows the effect of CBR on gas composition and H_2_ production. When CBR increases from 0.8 to 1.2, the volume fraction of H_2_ increases rapidly from 38.3% to 42.2%, and the volume fraction of CO decreases from 23.8% to 22.5%. CO_2_ also shows a decreasing trend, and the volume fraction decreases from 17.4% to 16.4%. The volume fractions of CH_4_ were relatively stable, maintained at about 16.5%. The increase of CBR increases the amount of H_2_ and decreases the amount of CO. it can be explained that according to le Chatelier’s principle if the partial pressure of the product is less than that of the reactant, the reaction will move forward. The catalyst b contains CaO, which can absorb CO_2_ and produce CaCO_3_. The occurrence of reaction R10 will promote the positive movement of reaction R9, so it consumes CO and produces more H_2_. If the CBR continues to increase from 1.2 to 1.4, the change tends to be gentle. It may be that the excess catalyst b fails to contact biomass particles, so it does not play a corresponding role. Therefore, there is an optimal value of 1.2 for the mass ratio of catalyst to biomass.

### 3.4 Effect of Temperature on Gasification

The selection of reaction temperature is very important to the gasification process. The gasification conditions are as follows: the CBR of catalyst b is 1.2, the particle size of biomass is less than 0.5 mm and the SBR is 1.0. The yield of each product (gas, tar, and char) and its effect on gas components were investigated when the reaction temperatures are 550, 600, 650, and 700°C respectively.

**FIGURE 10:**
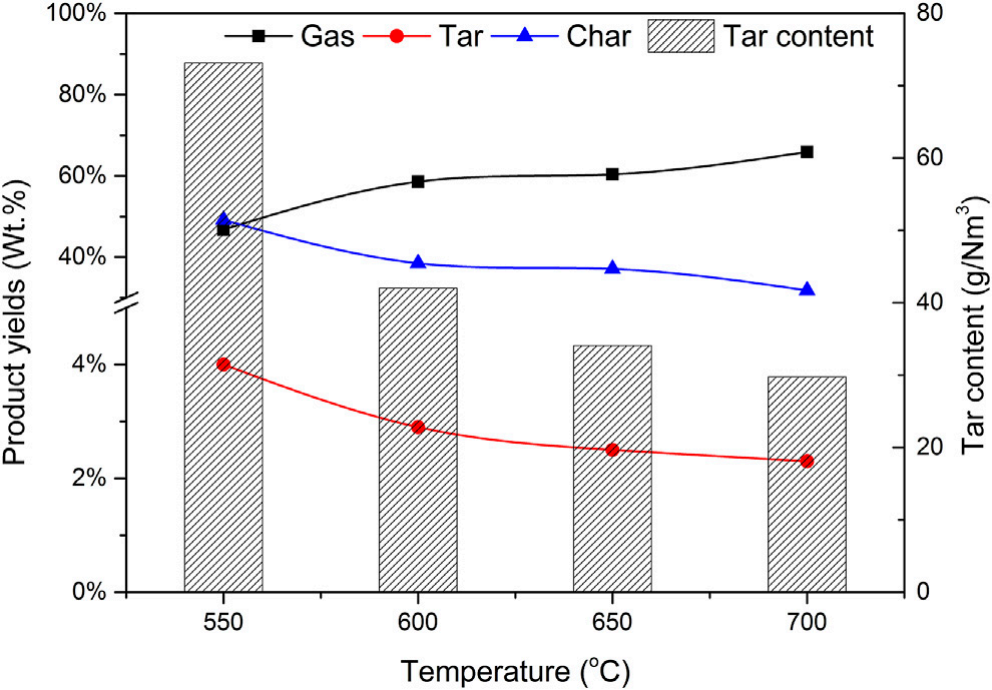
Effect of temperature on product yields and tar content.

Figure 10 shows the effect of reaction temperature on the components of each product and tar content. When the reaction temperature is 550°C, the proportion of gas in the product is 46.8%. When the temperature rises, the percentage of gas increases, and the peak value reaches 65.9% when the temperature rises to 700°C. This is because the increase of reaction temperature is conducive to biomass pyrolysis, gasification, and gas catalytic reforming, resulting in increased gas production. The yield of tar and char has the opposite trend with temperature because at higher furnace temperature, tar cracking and transfer reaction will take place further reactions, such as R2, R7, and R8, resulting in more non-condensable gas, which is consistent with the research results of Li et al. (Li et al., 2007). Therefore, as the temperature increases from 550 to 700°C, the total output of gas products increases significantly.

**FIGURE 11:**
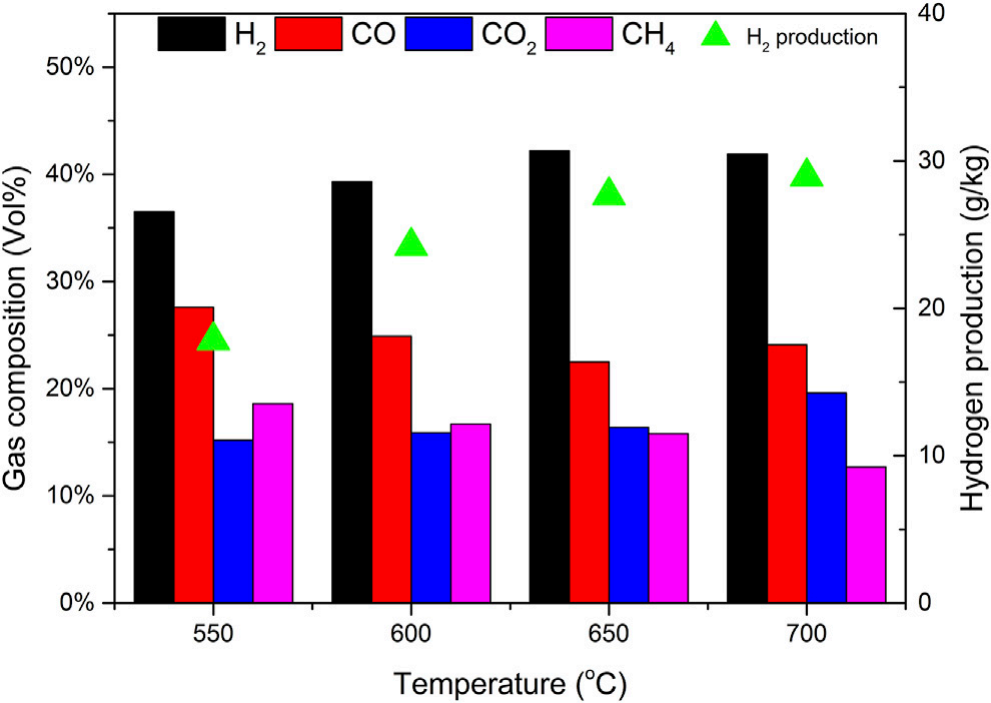
Effect of temperature on gas composition and hydrogen production.

The effect of reaction temperature on gas composition and H_2_ production is shown in Figure 11. The reaction temperature increases from 550 to 650°C, and the volume fraction of H_2_ increases from 36.5% to 42.2%. CO decreased from 27.6% to 22.5% and the volume fraction of CH_4_ decreased slightly from 18.6% to 15.8%. This is because when water vapor is introduced, the temperature rise is conducive to some reactions related to water vapor in the process of biomass catalytic reforming, such as hydrocarbon conversion reactions R4 and R5, water vapor conversion reactions R7, and R8, and carbon monoxide conversion reaction R9 in the direction of H_2_ generation. Therefore, with the increase of temperature, the content of H_2_ in the gas increases rapidly. The carbon monoxide shift reaction R9 will also proceed violently in the direction of generating H_2_. While the H_2_ content increases rapidly, the fraction of CO decreases with the increase of temperature, and the content of CO_2_ increases with the increase of temperature, from 15.2% to 16.4% gradually. However, the increase of temperature also promotes the reverse reaction of carbon dioxide reduction reaction R6 and reaction R10. Under the joint action of the three reactions, the increase of CO_2_ is small. When the temperature continues to rise to 700°C, the high temperature further intensifies the reverse reaction of R10, further increases the content of CO_2_ sharply, and even reduces the volume fraction of H_2_ in the gas. Considering comprehensively, it is most suitable to set the reaction temperature at 650°C and the production of hydrogen has reached 27.65 g/kg.

### 3.5 Effect of Water Vapor/Biomass Ratio (SBR) on Gasification

Steam is also an important variable of biomass catalytic gasification. Using catalyst b as the catalyst, CBR is 1.2, the gasification reaction temperature is 650°C, biomass particle size <0.5 mm, the effects of steam/biomass ratio (SBR) of 0.6, 0.8, 1.0, and 1.2 on gasification results were studied.

**FIGURE 12:**
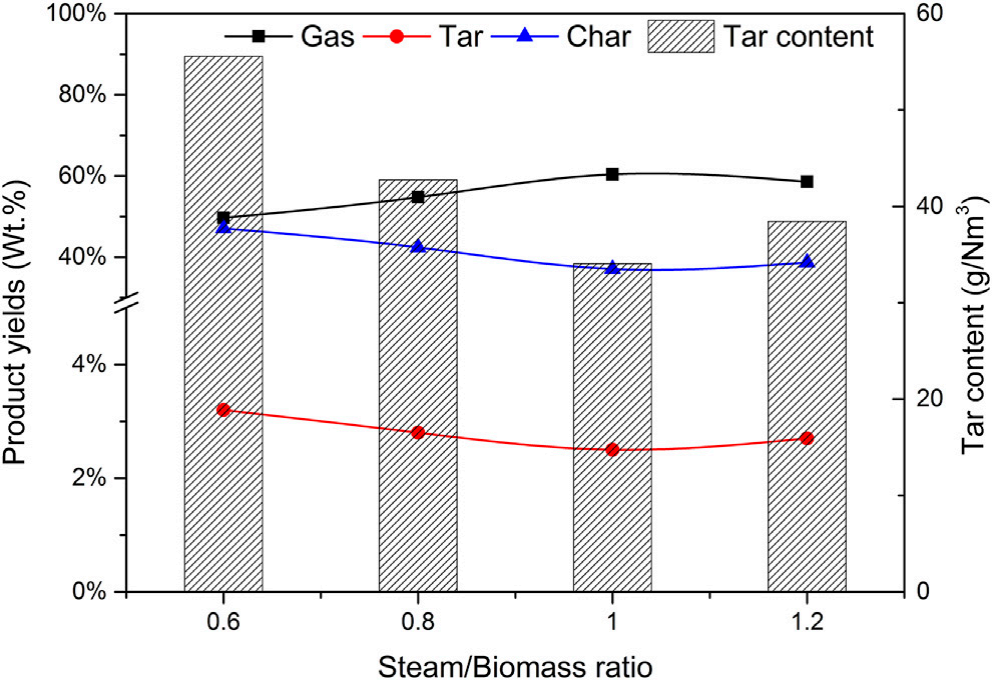
Effect of SBR on product yields and tar content.

Figure 12 depicts the effect of steam/biomass ratio (SBR) on the yield of pyrolysis reaction products and tar content. When SBR is less than 1.0, the gas yield increases with the increase of SBR, while the yield of tar and char shows the opposite trend. Wei et al. (Wei et al., 2007) found the same trend. This phenomenon can be attributed to the increased SBR, which provides more favorable conditions for tar cracking and carbon gasification. When SBR increased from 0.6 to 1.0, the gas yield increased from 49.7% to 60.4%, and the yields of tar and char decreased from 3.2% to 2.5% and 47.1.% to 37.1% respectively. These trends are highly consistent with other reports by researchers (Zhou et al., 2018). However, when too much steam is introduced and SBR is 1.2, the gas production rate decreases, which may be caused by the decrease of temperature in the furnace caused by the heat absorbed by excess water.

**FIGURE 13:**
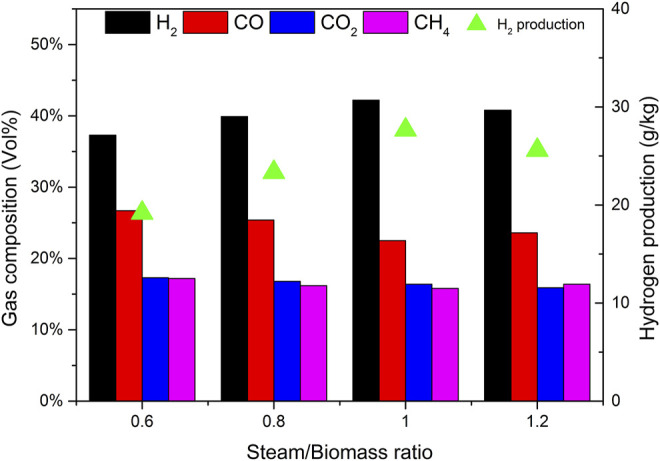
Effect of SBR on gas composition and hydrogen production.

As can be seen from Figure 13, when SBR increases from 0.4 to 0.8, the H_2_ content increases significantly, because the introduction of water vapor is conducive to tar cracking and char gasification, resulting in higher H_2_ production. According to Le Chatelier’s principle, the introduction of steam in the system will move the reactions R7, R8, and R9 in the positive direction to produce more hydrogen. When SBR is less than 1.0, the CO content decreases with the increase of water vapor, which is the direction of hydrocarbon conversion reactions R5 and R6, water vapor conversion reactions R8 and R9 to generate H_2_ and reduce CH_4_ and other hydrocarbons. At the same time, in the carbon monoxide shift reaction R9, due to the increase of the amount of water vapor and the acceleration of the positive reaction speed, the content of CO_2_ will increase, while the increased carbon dioxide will be absorbed by CaO in the catalyst. Therefore, in the process of increasing SBR, the content of CO_2_ will decrease, which is also confirmed by Acharya’s research (Acharya et al., 2010). It can be seen from Figure 13 that the addition of an appropriate amount of steam can improve the yield and quality of gas, but when the steam is excessive, the quality of gas begins to decline. There is an optimal value of SBR, which is 1.0 in this experimental study.

### 3.6 Effect of Biomass Particle Size on Gasification

**FIGURE 14:**
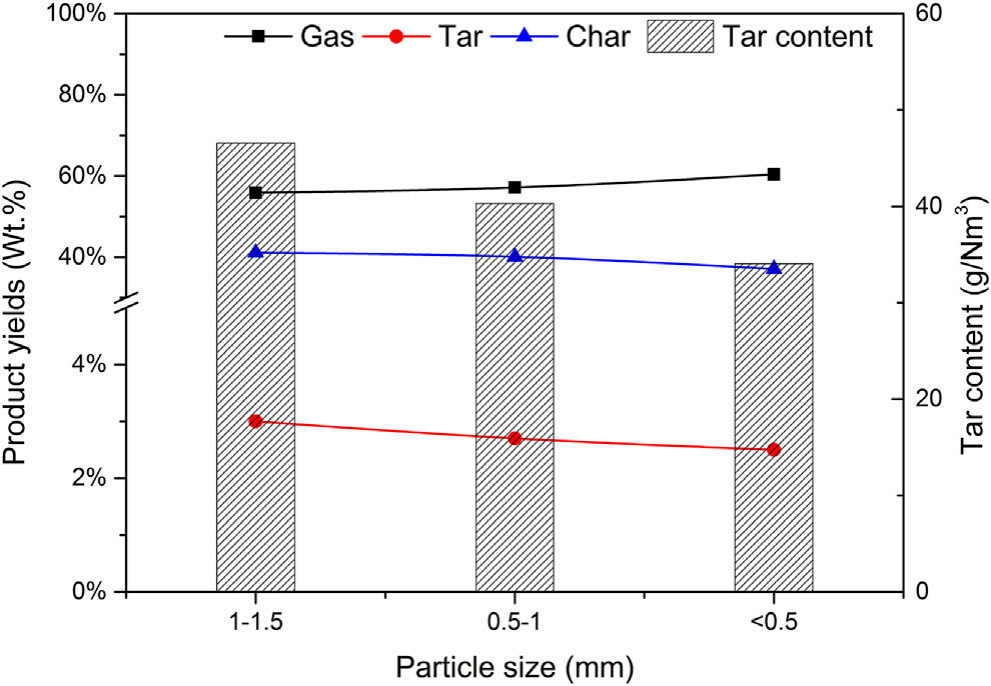
Effect of particle size on product yields and tar content.

Using catalyst b as the catalyst, CBR was 1.2, the gasification reaction temperature was 650°C and SBR was 1.0. The effects of biomass particle size <0.5, 0.5–1, and 1.0–1.5 mm on gasification results were studied. Figure 14 depicts the effect of biomass particle size on the yield of gasification products and tar content. As expected, with the increase of particle size, the gas yield decreases, while the yield of tar and char shows the opposite trend. These results are consistent with those obtained by Mohammed et al. (Mohammed et al., 2011). With the decrease of particle size, the surface area of biomass particles increases, which improves the heating rate of biomass particles and promotes the pyrolysis reaction of biomass. With the increase of biomass particle size, the heat transfer resistance increases, resulting in incomplete gasification and coking. Another reason is that the size of biomass particles affects the fluidization state in the gasifier. Under the same conditions, the pyrolysis diffusion of biomass particles is mainly controlled by the diffusion of a gas. Due to its large weight, larger particles are often difficult to diffuse and cannot effectively contact the catalyst, resulting in insufficient reaction R1 and low gasification degree (Zhang et al., 2007).

**FIGURE 15:**
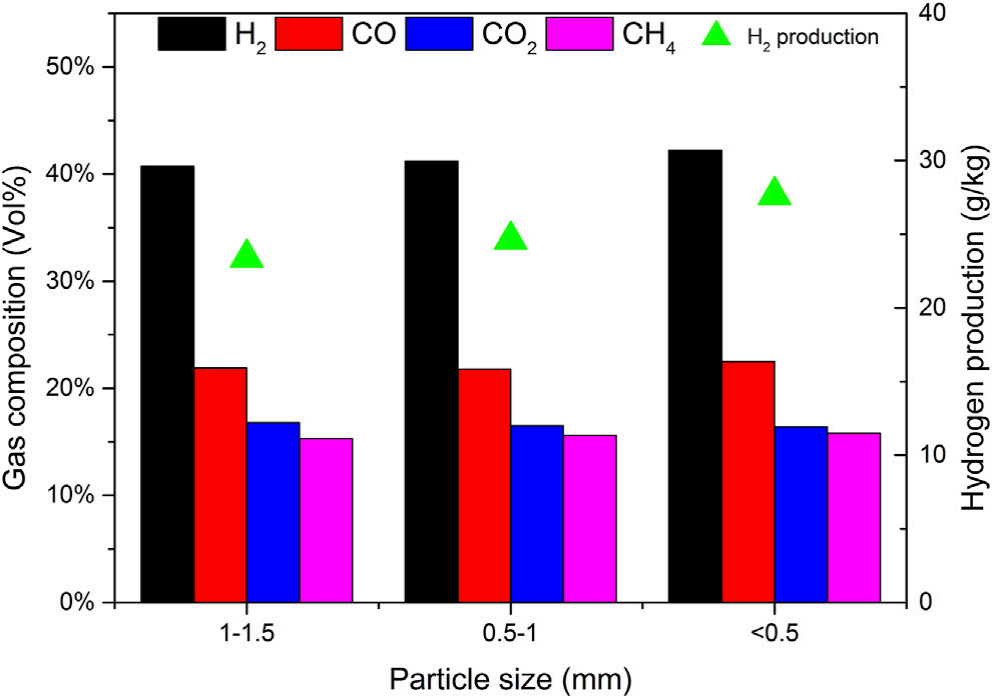
Effect of particle size on gas composition and hydrogen production.

Figure 15 shows the effect of biomass particle size on gas composition and H_2_ production. It can be seen that when the particle size decreases, the content of H_2_, CO, and CH_4_ increases, while the content of CO_2_ decreases. When the biomass particle size decreases, it is conducive to the full contact between biomass and water vapor, and the reactions R7 and R8 are enhanced. More CO_2_ is produced in the reaction process. Due to the existence of CaO in the catalyst, the biomass particles are small and in closer contact with the catalyst, which promotes the progress of reaction R10 and absorbs CO_2_ more fully. In general, the particle size of biomass material has a certain impact on the gas production characteristics of steam catalytic gasification. As the particle size of the material decreases, the H_2_ content in the generated gas increases. Compared with the influence of temperature and steam flux, the influence of material particle size is not very significant.

## 4 Conclusion

1) Three kinds of Fe-based composite catalysts with different loading were prepared. The analysis of catalytic activity for hydrogen production from biomass steam gasification showed that catalyst b with 10% Fe loading showed better activity than the other two catalysts.

2) XRD characterization shows that when the mass fraction of loading is 10%, Fe species are well dispersed on the carrier; N_2_ adsorption and desorption showed that catalyst b had a large specific surface area and pore structure; The TPR analysis of catalyst b shows that Fe species are successfully loaded on the composites. Several characterization methods show that catalyst b has the potential for high catalytic activity.

3) By changing the reaction conditions, the optimal reaction conditions were selected, CBR = 1.2, temperature 650°C, SBR = 1.0, particle size <0.5 mm. Under these conditions, the gas yield reached 60.4%, and the tar yield and char yield decreased to 2.5% and 37.1% respectively. The proportion of hydrogen in the gas composition has also reached a high level of 42.2% and the production of hydrogen has reached 27.65 g/kg. The tar content reaches the lowest value of 34.07 g/Nm^3^.

## Data Availability

The original contributions presented in the study are included in the article/Supplementary Material, further inquiries can be directed to the corresponding authors.
