# Supplementary material for: Application of Fe Based Composite Catalyst in Biomass Steam Gasification to Produce Hydrogen Rich Gas
Source: Front Chem. 2022 Apr 12;10:882787. doi: 10.3389/fchem.2022.882787 (PMC9039233; doi:10.3389/fchem.2022.882787)
Supplement: Supplementary file 1 [file Table1.DOCX]

***Supporting Information***

**Application of Fe based composite catalyst in biomass steam gasification to produce hydrogen rich gas**

Liang Zhou^1^, Zhiyong Yang^2^, Deju Wei^2^, Heng Zhang^3^*, Wei Lu^4^*

^1^School of Chemistry and Chemical Engineering, Guangxi University, Nanning 530004, China

^2^School of Chemical Engineering, Guizhou Institute of Technology, Guiyang 550003, China

^3^State Key Laboratory Breeding Base of Green Pesticide & Agricultural Bioengineering, Key Laboratory of Green Pesticide & Agricultural Bioengineering, Ministry of Education, State-Local Joint Laboratory for Comprehensive Utilization of Biomass, Center for Research & Development of Fine Chemicals, Guizhou University, Guiyang 550025, China

^4^School of Mechanical Engineering, Guangxi University, Nanning 530004, China

*Corresponding author: [hzhang23@gzu.edu.cn](mailto:hzhang23@gzu.edu.cn) (H Zhang); [luwei@gxu.edu.cn](mailto:luwei@gxu.edu.cn) (W Lu).

**Table S1** Proximate analysis and Ultimate analysis of pine sawdust

| Proximate analysis（wt%） | | Ultimate analysis（wt%） | |
| --- | --- | --- | --- |
| Moisture | 9.23 | C | 48.53 |
| Volatile | 72.50 | H | 6.25 |
| Fixed carbon | 16.19 | O | 44.60 |
| Ash | 2.08 | N | 0.58 |
|  |  | S | 0.04 |
